# Breaking down biocentrism: two distinct forms of moral concern for nature

**DOI:** 10.3389/fpsyg.2014.00905

**Published:** 2014-08-20

**Authors:** Joshua Rottman

**Affiliations:** Department of Psychological and Brain Sciences, Boston UniversityBoston, MA, USA

**Keywords:** environmentalism, moral psychology, biocentrism, ecocentrism, harm, purity

Why should deforestation be stopped? Why should greenhouse gas emissions be reduced? To answer moral queries such as these, one could point to the wellbeing of future generations and the survival of the human species. One could also appeal to the preservation of biodiversity and the intrinsic value of the natural world. These two attitudes are indeed distinct, and many scholars have therefore differentiated between “anthropocentric” (also called “homocentric” or “altruistic”) and “biocentric” (also called “ecocentric” or “biospheric”) concerns for the environment (e.g., Kahn and Friedman, 1995; Howe et al., 1996; Kahn, 1997, 2006; Schultz and Zelezny, 1998; Severson and Kahn, 2010; Hussar and Horvath, 2011; Steg and de Groot, 2012). Anthropocentric concerns for the environment are narrowly aimed at preserving the welfare of humans, while biocentric concerns are oriented toward protecting non-human organisms and nature as a whole. While anthropocentrism can sometimes lead to pro-environmental attitudes and actions, biocentrism is more reliably and robustly related to environmentalism, both for abstract values and for concrete behaviors (e.g., Gagnon Thompson and Barton, 1994; Schultz et al., 2005; Steg et al., 2005; de Groot and Steg, 2008). This makes sense, as anthropocentrism promotes the preservation of the environment as a means to an end rather than an end in itself. However, biocentrism treats environmentalism as a moral imperative independently of its impact on human flourishing.

In order to promote environmentalism, it is crucial to understand how moral intuitions can be made to resonate with values related to preserving the natural world (Markowitz and Shariff, 2012). Therefore, examining the psychological foundations of biocentrism promises to illuminate a path toward a more sustainable future. For this goal to be achieved, the idea of biocentrism must be deconstructed and operationalized in psychologically meaningful terms. In particular, biocentrism is unlikely to be a singular stance; rather, it plausibly consists of at least two qualitatively distinct attitudes. First, biocentrism can stem from a desire to *avoid hurting sentient beings* (e.g., harboring concerns about killing animals). Second, biocentrism can stem from a desire to *uphold purity* in nature (e.g., harboring concerns about violating the sanctity or telos of natural kinds). Avoiding harm and preserving purity have been identified as two separate forms of moral concern that rely on functionally distinct systems of cognitive and emotional processing (e.g., Rozin et al., 1999; Haidt and Joseph, 2004; Young and Saxe, 2011; Graham et al., 2013). Therefore, the concept of biocentrism potentially obscures a psychologically important distinction in environmentalist attitudes.

Subdividing biocentrism into two separate moral concerns—about harm and about purity—provides a meaningful starting point for investigating its psychological underpinnings (Rottman et al., in press). Understanding biocentrism in terms of avoiding harm emphasizes the importance of extending mental states and rights to non-human entities. In particular, the tendency toward anthropomorphization can enhance environmentalism because non-humans are conceptualized as possessing more humanlike minds, thus having a heightened capacity to be harmed (Waytz et al., 2010). Multiple studies have demonstrated that anthropomorphizing other species or nature as a whole increases biocentric beliefs and behaviors (e.g., Bastian et al., 2012; Butterfield et al., 2012; Tam et al., 2013). Additionally, taking the perspective of animals that are being harmed leads to greater biocentric concerns for the environment (Schultz, 2000). Biocentric concerns about harming nature therefore rest on expanded capacities for person perception and subjective ascriptions of others' suffering (Gray et al., 2012), such that the scope of justice is expanded to include non-human beings. In this way, biocentrism can arise from the same psychological processes that produce anthropocentrism; the only difference is that they are applied to a broader moral circle. This could explain why biocentrism and anthropocentrism are sometimes found to overlap (e.g., Stern and Dietz, 1994).

Alternatively, biocentrism is sometimes rooted in concerns about purity or sanctity. In particular, nature can be conceptualized as a divine creation that people have a sacred duty to preserve (Wardekker et al., 2009), and this sanctification of the planet has been shown to increase pro-environmental beliefs and behaviors (e.g., Tarakeshwar et al., 2001). This purity-based construal may be especially salient for particular populations. For example, framing environmental messages in terms of upholding the purity of the environment increases the pro-environmental attitudes of political conservatives, while harm-based framings do not exert any effect (Feinberg and Willer, 2013). Additionally, although this form of biocentrism is probably predominant in religious and spiritual individuals (Sherkat and Ellison, 2007), it is likely found in secular individuals as well. Indeed, sanctification often occurs outside of theistic settings (Pargament and Mahoney, 2005), and the treatment of certain aspects of nature as sacred may stem from a more general deontological tendency to harbor “protected values” (Baron and Spranca, 1997). Therefore, biocentrism is sometimes orthogonal to considerations about harm, arising from very different psychological processes than those that produce anthropocentric concerns.

In sum, biocentrism can be driven by at least two distinct moral concerns. When biocentrism is focused on avoiding harm, it is primarily geared toward protecting sentient and humanized entities, and it is likely moderated by individual differences in the tendency to anthropomorphize nature. Conversely, when biocentrism is focused on upholding the purity of the environment, it primarily operates at a more systemic level rather than focusing on the protection of discrete, individuated entities. Additionally, a purity-based biocentrism is likely moderated by individual differences in spirituality and in tendencies to treat certain objects as possessing inherent value. The psychological profiles underlying biocentric environmentalist attitudes due to harm concerns and due to purity concerns are therefore very different, although they might sometimes co-occur. Recognizing this distinction carries substantial implications for the efficacy of particular forms of environmentalist discourse (Rottman et al., in press). An adequate account of environmentalist attitudes requires that the construct of biocentrism is ultimately replaced by more nuanced distinctions. Understanding this aspect of human psychology will serve as a crucial step in putting an end to deforestation, greenhouse gas emissions, and countless other environmental threats.

## Conflict of interest statement

The author declares that the research was conducted in the absence of any commercial or financial relationships that could be construed as a potential conflict of interest.
